# Aberrations in Peripheral Blood Neuron‐Specific Enolase Levels in Depression: A Systematic Review and Meta‐Analysis

**DOI:** 10.1155/da/7759663

**Published:** 2026-07-13

**Authors:** Qianbing Ge, Yangke Li, Tianye Sun, Yongbo Tan, Xingru Wu, Chuanyu Wu, Jiao Zou, Ling Yang, Jingxuan Zhang, Junwei Gao

**Affiliations:** ^1^ Department of Military Cognitive Psychology, School of Psychology, Third Military Medical University (Army Medical University), Chongqing, 400038, China, tmmu.edu.cn; ^2^ College of Basic Medical Sciences, Third Military Medical University (Army Medical University), Chongqing, 400038, China, tmmu.edu.cn; ^3^ Psychological Training Center, School of Psychology, Third Military Medical University (Army Medical University), Chongqing, 400038, China, tmmu.edu.cn; ^4^ Department of Emergency, Southwest Hospital, Third Military Medical University (Army Medical University), Chongqing, 400038, China, tmmu.edu.cn; ^5^ School of Psychology, Third Military Medical University (Army Medical University), Chongqing, 400038, China, tmmu.edu.cn

**Keywords:** major depressive disorder, meta-analysis, neuron-specific enolase, poststroke depression, serum

## Abstract

**Purpose:**

Neuron‐specific enolase (NSE) is a type of glycolytic enzyme enolase. As NSE is predominantly localized in endocrine cells and neurons, it is frequently recognized as a specific biomarker for peripheral neuroendocrine cells and neurons. Recently, numerous clinical studies have investigated the correlation between peripheral blood NSE levels and depression; however, the findings remain inconsistent. The objective of this study is to systematically evaluate peripheral blood NSE levels in patients with various types of depression.

**Methods:**

We conducted an extensive literature review using various databases such as PubMed, Embase, Cochrane Library, Web of Science, Wanfang, and China National Knowledge Infrastructure (CNKI). A random‐effects model was utilized to compute the pooled effect size (ES) (Hedges’ *g*). Sensitivity analysis, assessment of publication bias, and subgroup analysis were conducted using Comprehensive Meta‐Analysis software version 3.0 (CMA 3.0) software.

**Results:**

A total of 1175 records were screened for initial eligibility, and 18 reports reported in 18 studies that met the inclusion criteria were included in the meta‐analysis. The primary analysis revealed that abnormally elevated levels of NSE in peripheral blood are closely related to the risk of occurrence of major depressive disorder (MDD) or poststroke depression (PSD). Analysis results from different groups on MDD or PSD showed similar outcomes. Sensitivity analysis confirmed the robustness of our findings. The findings on publication bias suggested that this study did not exhibit any significant publication bias.

**Conclusions:**

The findings of our study suggest that abnormally elevated NSE levels in peripheral blood are closely related to the risk of depression, especially MDD and PSD. These findings offer valuable references for the monitoring and early warning of depression. However, the applicability to other subtypes of depression and the clinical translational value requires further validation. In the future, clinical studies with more participants are necessary.

## 1. Introduction

Depression is a mood disorder marked by ongoing sadness and/ or anhedonia, leading to significant impairments in daily functioning [1]. Major depressive disorder (MDD), a highly prevalent subtype of clinical depression, is marked by persistent depressive mood, lack of interest, and additional symptoms like sleep disturbances and significant weight changes, according to the Diagnostic and Statistical Manual of Mental Disorders‐5 (DSM‐5). Poststroke depression (PSD) refers to a mood disorder that arises after a stroke, presenting with depressive features, major depressive‐like episodes, or a combination of mood characteristics [2]. Therefore, PSD encompasses the symptoms associated with MDD. Clinically, PSD is a form of secondary depression, presenting with symptoms of enduring emotional distress, cognitive impairment, and somatic complaints. PSD is also a relatively frequent complication after stroke and can affect one‐third of patients surviving a stroke. PSD can occur at any stage of stroke, but there is a peak incidence of PSD within the first year of stroke (about one‐third) [3]. Globally, the incidence of depression is increasing year after year, with about 300 million individuals suffering from MDD [4]. Depression is one of the top contributors to disease burden [5], disability, and loss of life expectancy worldwide [6]. WHO statistics indicate that around 700,000 people commit suicide each year, with depression being a major cause. Depression, therefore, has become an important issue for mental illness disorders. To date, the pathophysiology of depression is incompletely understood, and the diagnosis tools and treatment are nonexistent. The lack of objective diagnostic biomarkers makes the assessment and diagnosis of depression subjective and frequently misdiagnosed. Consequently, identifying more biomarkers is essential for better early detection and intervention in depression.

The pathophysiology of depression is highly complex. It has been widely acknowledged that MDD and PSD are associated with multiple pathogenic factors. The currently well‐established theories of pathogenesis include the neurotransmitter and receptor hypothesis (e. g., the traditional monoamine theory), the hypothalamic–pituitary–adrenal (HPA) axis hypothesis, the cytokine hypothesis, and the genetic and epigenetic abnormality hypothesis [7, 8]. In recent years, several studies have indicated that changes in the levels of relevant cytokines, particularly inflammatory factors, play a significant role in the development of depression and cognitive impairment [9]. Specifically, the secretion of numerous inflammatory factors can induce a range of processes, including neuroinflammation, neurodegeneration, neuroendocrine dysfunction, and impaired neuron and glial cell functions [10, 11]. Therefore, some markers of neural damage may provide important references for the early diagnosis and treatment of depression.

Neuron‐specific enolase (NSE), which is part of the enolase superfamily, is predominantly found in neurons and neuroendocrine cells. It plays a critical role in catalyzing a key reaction in the glycolytic pathway: the conversion of 2‐phosphoglycerate into phosphoenolpyruvate, which serves as an essential substrate for the cellular energy metabolism [12, 13]. Moreover, NSE contributes to neuronal development, synapse formation, and cell cycle regulation. Notably, under physiological conditions, NSE is not secreted. Neuronal damage causes NSE to be released from the cytoplasm into the cerebrospinal fluid (CSF), after which it enters the bloodstream. Consequently, an elevation in peripheral NSE levels may serve as an indicator of structural neuronal damage [14, 15]. In recent years, NSE has been shown to be effective in evaluating the prognosis of seizures, traumatic brain injury, and comatose patients [16–18]. More significantly, NSE has also been investigated in relation to neurological and psychiatric disorders. A meta‐analysis on Alzheimer’s disease (AD) revealed that the levels of NSE in the CSF of patients were considerably higher than those of the control group, which demonstrated the potential of NSE as an objective biomarker for AD [19]. Another study on bipolar disorder found that the level of NSE fluctuates with disease progression and treatment stage [20]. Over the previous decade, various research efforts have examined the association between peripheral NSE and depression, but the outcomes have been inconsistent. For this reason, we conducted this meta‐analysis to explore the association between peripheral NSE and depression further.

## 2. Materials and Methods

### 2.1. Publication Search

As of March 19, 2025, a systematic search was conducted across six databases —PubMed, Embase, Cochrane Library, Web of Science, Wanfang, and China National Knowledge Infrastructure (CNKI). The retrieval keywords were as follows: “depress,” “depressive,” or “depression;” and “Neuron‐specific enolase,” “Neuronal enolase,” “Gamma‐enolase,” or “neuron specific enolase.” Concurrently, we performed citation tracking through reference lists of retrieved articles to supplement the search and identify additional potentially eligible studies.

### 2.2. Inclusion and Exclusion Criteria

The present meta‐analysis was performed in accordance with the Preferred Reporting Items for Systematic Reviews and Meta‐Analyses ( PRISMA ) guidelines. The studies considered for inclusion had to fulfill specific criteria as follows: (1) clinical studies quantifying peripheral blood NSE concentrations in depression patients and controls and (2) reporting of NSE levels as mean ± standard deviation (SD). We excluded studies that exhibited any of the following characteristics: (1) studies lacking adequate data; (2) nonresearch publication types (e. g., case reports, and commentaries); (3) non–full‐text publications (e. g., conference abstracts); and (4) experimental animal research.

### 2.3. Data Extraction

Based on predetermined criteria for inclusion and exclusion, two investigators independently screened the records that were retrieved. Data extraction was performed using Excel, including author name, year of publication, country, diagnostic criteria, source of patients, NSE assay methods, type of sample, age and gender of participants, sample size, mean, SD, and *p*‐ value . All studies fully reported peripheral blood NSE concentration data, which were presented as mean and SD; therefore, no data imputation was performed. Any discrepancies during screening or extraction were resolved through consensus discussions.

### 2.4. Statistical Analysis

The primary outcome was to investigate the difference in peripheral blood NSE levels between patients with depression and control groups. To measure effect size (ES) with less bias, Hedges’ *g* was calculated using sample sizes, mean NSE levels, corresponding SDs, and *p*‐ values . The association between peripheral blood NSE levels and depression was quantified using the pooled Hedges’ *g* with 95% confidence intervals (CIs). Heterogeneity across studies was evaluated via the *I* ^2^‐value and Cochrane’s *Q*‐ test . A random‐effects model was chosen when significant heterogeneity was present (*Q* statistic *p* < 0.10 or *I* ^2^ > 50%); otherwise, a fixed‐effects model was applied. Subgroup analyses utilizing various control types were performed to examine the sources of heterogeneity. Sensitivity analyses were conducted by sequentially removing individual studies to evaluate their impact on the overall results. Publication bias was evaluated qualitatively by examining the symmetry of a funnel plot and quantitatively using Egger’s test. In the present study, *p* < 0.05 was considered statistically significant. All statistical analyses were executed using Comprehensive Meta‐Analysis version 3 software.

## 3. Results

### 3.1. Characteristics of Eligible Studies

**Figure 1:**
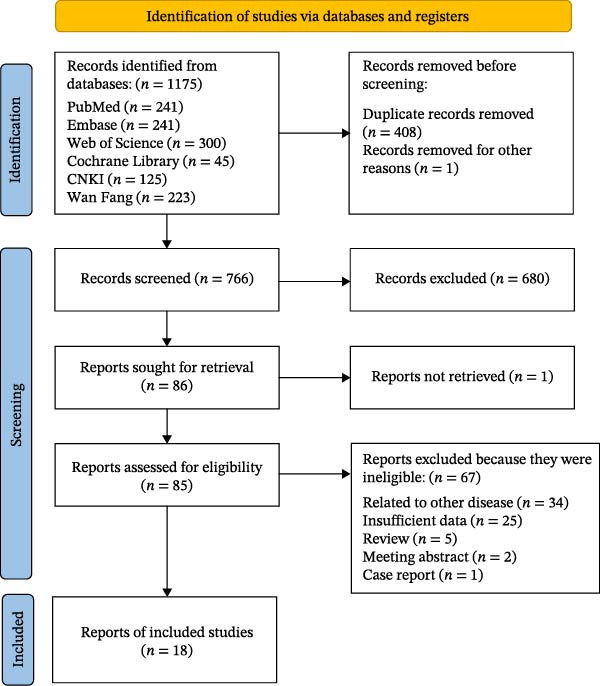
Study selection flow diagram. The flowchart illustrates the search strategy and the process of study selection.

Our preliminary investigation resulted in 1175 records obtained through various search term combinations on PubMed (241 records), Embase (241 records), Web of Science (300 records), Cochrane Library (45 records), CNKI (125 records), and Wan Fang (123 records). Among these, 408 records were excluded due to duplication. Following the screening of titles and abstracts, 680 records were excluded due to mismatches. Upon full‐text reading, 67 records were removed: 34 pertaining to other diseases, 25 with inadequate data, 5 being reviews, 2 being meeting abstracts, and 1 being a case report. Finally, our meta‐analysis encompassed 18 studies (18 reports) [21–38]. The flow diagram can be seen in Figure 1.

All NSE measurements in the included studies were exclusively derived from serum samples, and the data were completely extracted and reported as the mean ± SD. Geographically, the studies originated predominantly from Turkey, Brazil, Germany, and China. The diagnostic reference standards for depression were primarily based on the DSM or Chinese Classification and Diagnostic Criteria of Mental Disorders (CCMDs). In total, we collected nine studies with MDD and nine studies with PSD as experimental groups, respectively. Notably, all PSD study participants were Chinese. The included studies’ characteristics are detailed in Table 1.

**Table 1:** Characteristics of the studies included in the meta‐analysis.

First author and publication year	Country	Diagnostic criteria	Source	Detection method	Sample size		Cases				Controls				*p*
					Case	Control	Type	Age (years)	Gender (M/ F)	Mean ± SD (ng/ mL)	Type	Age (years)	Gender (M/ F)	Mean ± SD (ng/ mL)	
Kozak [23] 2019	Turkey	DSM‐IV	Necmettin Erbakan University in the Meram Faculty of Medicine in Konya, Turkey	ELISA	17	36	MDD	67.47 ± 11.2	8/9	4.47 ± 2.71	AIS	65.25 ± 13.98	19/17	6.19 ± 6.29	0.32
Uysal [29] 2025	Turkey	DSM‐5	Ethics Committee of Ankara Yildirim Beyazit University Yenimahalle Education and Research Hospital	ECLIA	43	40	MDD	NA	3/40	14.89 ± 4.94	Healthy	NA	3/37	14.81 ± 3.29	>0.05
Güleş [22] 2020	Turkey	DSM‐IV	Kocaeli University, Turkey	ELISA	10	10	MDD	42.3 ± 9.0	5/5	75.41 ± 34.45	Healthy	42.1 ± 8.6	5/5	18.10 ± 16.39	0.007
Wiener [33] 2013	Brazil	DSM‐IV	Hospital São Francisco de Paula, Universidade Católica de Pelotas, Pelotas, Brazil	ECLIA	36	36	MDD	27.39 ± 4.72	29/7	2.19 ± 1.78	Healthy	27.58 ± 5.00	30/6	3.55 ± 2.19	0.004
Schroeter [28] 2008	Germany	DSM‐IV, ICD‐10	NA	NA	10	10	MDD	48.8 ± 16.9	8/2	7.41 ± 2.18	Healthy	45.1 ± 8.9	7/3	5.91 ± 2.24	>0.05
Li [25] 2017	China	DSM‐IV	Yan’an University Xianyang Hospital	ELISA	40	40	MDD	42.2 ± 3.8	18/22	11.98 ± 2.69	Healthy	41.8 ± 3.2	20/20	4.52 ± 0.95	<0.001
Wang [30] 2017	China	CCMD‐3	The 1st People Hospital in Guiyang	ELISA	82	16	MDD	60.4 ± NA	42/40	14.79 ± 3.85	Healthy	60.2 ± NA	8/8	7.85 ± 2.05	0.002
Yang [35] 2010	China	CCMD‐3	The First Affiliated Hospital of Nanchang University	ECLIA	25	25	MDD	25.16 ± 6.69	11/14	15.78 ± 6.12	Healthy	24.68 ± 7.0	11/14	9.20 ± 5.49	<0.01
Zheng [37] 2012	China	CCMD‐3	The Affiliated Hospital of Guiyang Medical College	ELISA	31	25	MDD	41.77 ± 12.7	13/18	11.32 ± 3.19	Healthy	37.88 ± 10.75	10/15	4.53 ± 1.04	<0.01
Zhao [36] 2015	China	DSM‐IV	Zhengzhou University Affiliated Zhengzhou Central Hospital English	ELISA	42	42	PSD	68.2 ± 8.0	15/27	2.59 ± 1.48	AIS	69.0 ± 10.2	15/27	2.27 ± 1.84	0.292
Wang [31] 2018	China	CCMD‐3	Jilin Provincial Vanguard Hospital Neurointerventional Center	NA	60	60	PSD	57.1 ± 12.9	36/24	34.41 ± 3.51	AIS	57.4 ± 13.1	37/23	30.51 ± 3.75	<0.05
Wei [32] 2024	China	CCMD‐3	Yuncheng Central Hospital Affiliated to Shanxi Medical University	NA	32	69	PSD	58.79 ± 9.51	18/14	32.48 ± 5.36	AIS	57.01 ± 9.23	45/24	28.49 ± 5.08	<0.001
Li [26] 2023	China	CCMD‐3	Henan Provincial People’s Hospital	ECLIA	62	33	PSD	61.37 ± 9.32	38/24	33.11 ± 8.71	AIS	62.48 ± 8.66	18/15	16.01 ± 2.68	<0.001
Chen [21] 2021	China	NA	Luohe Third People’s Hospital	ELISA	128	172	PSD	51.28 ± 8.42	74/54	17.69 ± 3.24	AIS	52.17 ± 8.51	102/70	11.51 ± 2.78	<0.05
Meng [27] 2018	China	CCMD‐3	Department of Neurology, Sixth Affiliated Hospital of Sun Yat sen University	ELISA	46	90	PSD	61.0 ± 8.5	27/19	23.63 ± 7.62	AIS	63.0 ± 8.5	53/37	19.44 ± 5.38	0.001
Xie [34] 2021	China	CCMD‐3	Changde First People’s Hospital	ELISA	40	50	PSD	60.12 ± 6.76	22/18	32.03 ± 6.45	Healthy	61.09 ± 6.71	26/24	9.45 ± 2.89	<0.05
Lei [24] 2014	China	CCMD‐3	Luoyang Fifth People’s Hospital	ECLIA	89	50	PSD	53.14 ± 8.72	49/42	31.24 ± 10.56	Healthy	54.21 ± 5.23	31/19	13.87 ± 6.86	<0.05
Zhou [38] 2024	China	CCMD‐3	Jinhua Guangfu Oncology Hospital	ELISA	44	62	PSD	63.48 ± 6.59	26/18	7.35 ± 2.99	CAIS	63.45 ± 7.45	36/26	5.06 ± 2.36	<0.001

Abbreviations: AIS, acute ischemic stroke; CAIS, cancer‐associated ischemic stroke; ECLIA, electrochemiluminescence immunoassay; ELISA, enzyme‐linked immunosorbent assay; MDD, major depressive disorder; NA, not available; PSD, poststroke depression.

### 3.2. Main Association of MDD With NSE Levels in Peripheral Blood

#### 3.2.1. Quantitative Data Synthesis

**Figure 2:**
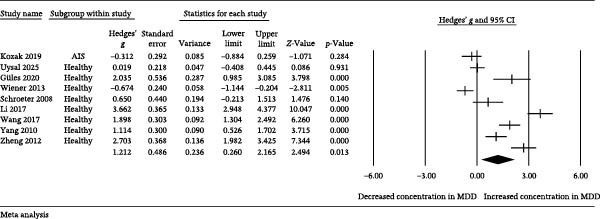
Forest plot of primary analysis on MDD. The overall effect estimate for the MDD showed significantly elevated NSE levels. Each black box represents a study’s point estimate, and the horizontal line shows the 95% CI. The diamond represents the pooled effect size (Hedges’ *g*). The vertical line at 0 represents the line of no effect; if the horizontal line of a study crosses this line, the effect of that study is not statistically significant. A Hedges’ *g*‐ value and its 95% CI entirely greater than 0 indicates higher NSE levels in the MDD group compared with controls (i. e., increased concentration in MDD), whereas values less than 0 indicate the opposite direction. CI, confidence interval; MDD, major depressive disorder; NSE, neuron‐specific enolase.

In our meta ‐analysis, we calculated the pooled ES and the 95% CI of nine studies in patients with MDD. We applied a random‐effects model to our analysis because of considerable heterogeneity of these studies (*I* ^2^ = 95.382, *p* < 0.001). Overall comparison indicated that patients with MDD had significantly higher levels of NSE in peripheral blood compared to controls (Hedges’ *g* = 1.212, 95% CI: 0.260–2.165, *p* = 0.013) (Figure 2). To analyze the source of the heterogeneity, a subgroup analysis based on healthy controls was performed first. This analysis revealed an increase in heterogeneity (*I* ^2^ = 91.248, *p* < 0.001). Despite this, the subgroup still exhibited a significant association between higher NSE levels and MDD (Hedges’ *g* = 1.408, 95% CI: 0.368–2.448, *p* = 0.008) (Supporting Information Figure S1) (Table 2). As for the national subgroup analysis, we found that in the Chinese subgroup, the NSE levels in the peripheral blood of MDD patients showed a significant difference (Hedges’ *g* = 2.329, 95% CI: 1.267–3.391, *p* < 0.001) (Supporting Information Figure S2). In contrast, in the studies from Turkey, the difference was not significant (Hedges’ *g* = 0.461, 95% CI: −0.543–1.465, *p* = 0.368) (Supporting Information Figure S3). However, whether Turkey (*I* ^2^ = 86.827, *p* = 0.001) or China (*I* ^2^ = 90.643, *p* < 0.001), the heterogeneity has not decreased significantly. In terms of assay methods, a significant difference was detected in the enzyme‐linked immunosorbent assay ( ELISA ) subgroup (Hedges’ *g* = 1.988, 95% CI: 0.551–3.425, *p* = 0.007) (Supporting Information Figure S4) but not in the electrochemiluminescence immunoassay ( ECLIA ) subgroup (Hedges’ *g* = 0.137, 95% CI: −0.795−1.070, *p* = 0.773) (Supporting Information Figure S5). Nevertheless, both ELISA (*I* ^2^ = 95.283, *p* < 0.001) and ECLIA (*I* ^2^ = 90.779, *p* < 0.001) subgroups continued to exhibit high heterogeneity. Additionally, no significant difference in peripheral blood NSE levels was found between MDD patients diagnosed using DSM‐IV criteria and controls (Hedges’ *g* = 1.054, 95% CI: −0.580−2.687, *p* = 0.206) (Supporting Information Figure S6), whereas a significant difference emerged when applying CCMD‐3 criteria (Hedges’ *g* = 1.885, 95% CI: 1.016–2.754, *p* < 0.001) (Supporting Information Figure S7). However, neither the former (*I* ^2^ = 96.531, *p* < 0.001) nor the latter (*I* ^2^ = 82.391, *p* = 0.003) subgroup analysis identified the source of heterogeneity.

**Table 2:** Summary of meta‐analysis results.

Groups	Studies (*n*)	Case (*n*)	Control (*n*)	Tests of association				Tests of heterogeneity		
				Model	Hedges’ *g* (95% CI)	*Z*	*p*‐ Value	*Q*‐ value	*p*‐ Value	*I* ^2^ (%)
MMD										
Total	9	294	238	RE	1.212 (0.260–2.165)	2.494	0.013	173.255	<0.001	95.383
Subgroups										
Healthy	8	277	202	RE	1.408 (0.368–2.448)	2.653	0.008	156.863	<0.001	95.538
ECLIA	3	104	101	RE	0.137 (−0.795 to 1.070)	0.289	0.773	21.689	<0.001	90.779
ELISA	5	180	127	RE	1.988 (0.551–3.425)	2.712	0.007	84.795	<0.001	95.283
China	4	178	106	RE	2.329 (1.267–3.391)	4.297	< 0.001	32.062	<0.001	90.643
Turkey	3	70	86	RE	0.461 (−0.543 to 1.465)	0.899	0.368	15.182	0.001	86.827
DSM‐IV	5	113	132	RE	1.054 (−0.580 to 2.687)	1.264	0.206	115.321	<0.001	96.531
CCMD‐3	3	138	66	RE	1.885 (1.016–2.754)	4.250	< 0.001	11.358	0.003	82.391
PSD										
Total	9	543	628	RE	1.564 (0.938 to 2.191)	4.896	< 0.001	167.125	<0.001	95.213
Subgroups										
AIS	6	370	466	RE	1.181 (0.517–1.844)	3.489	< 0.001	87.854	<0.001	94.309
Healthy	2	129	100	RE	3.226 (0.461–5.992)	2.286	0.022	38.324	<0.001	97.391
ECLIA	2	151	83	RE	2.061 (1.564–2.557)	8.138	< 0.001	2.212	0.137	54.792
ELISA	5	300	416	RE	1.643 (0.581–2.705)	3.032	0.002	139.102	<0.001	97.124

*Note*: *p*‐ Values were determined using random effects modeling in each systematic review and meta‐analysis. Between‐study heterogeneity was assessed using the Hedges’ *g* statistic, where *p* < 0.05 is considered statistically significant, and quantified by the *I* ^2^ statistic, where *I* ^2^ ≥ 50% is considered evidence of substantial heterogeneity.

Abbreviations: FE, fixed‐effect model; RE, random‐effect model.

These findings suggest that regional population factors, assay methods, and diagnostic criteria may contribute to the inconsistency across studies. Nevertheless, due to the persistently high heterogeneity within all subgroups, we should interpret the results more cautiously. Unfortunately, none of the subgroup analyses have reasonably explained the source of heterogeneity (Table 2).

#### 3.2.2. Sensitivity Analysis

To determine the influence of individual studies on the pooled ES, a sensitivity analysis was carried out by sequentially excluding each study. The significant association remained largely unaffected, indicating the robustness of our findings (Supporting Information Figure S8). Furthermore, our meta‐analysis demonstrated that no individual study could notably influence the heterogeneity among the studies. These findings further support the reliability of our study.

#### 3.2.3. Publication Bias

**Figure 3:**
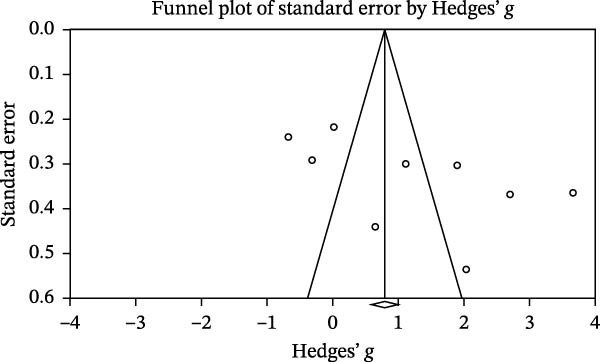
Funnel plot of precision using Hedges’ *g* statistics on MDD. Each dot represents an individual study included in the meta‐analysis. The vertical line represents the pooled effect estimate from the meta‐analysis. The inverted dashed lines outline the expected 95% confidence interval (CI) for the distribution of studies in the absence of bias. The symmetrical distribution of various studies on MDD patients suggests no evident publication bias.

A visual examination of the funnel plot suggested that there was no significant publication bias in the meta‐analysis (Figure 3). This observation was further supported by the Egger test, which yielded a *p*‐ value of 0.065. Additionally, the classic fail‐safe N method indicated that 208 missing studies would be necessary to reduce the *p*‐ value below 0.05. Consequently, it can be inferred that the publication bias did not significantly impact our findings.

### 3.3. Main Association of PSD With NSE Levels in Peripheral Blood

#### 3.3.1. Quantitative Data Synthesis

**Figure 4:**
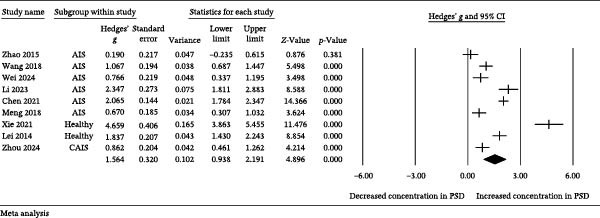
Forest plot of primary analysis on PSD. The overall effect estimate for the PSD showed significantly elevated NSE levels. Each black box represents a study’s point estimate, and the horizontal line shows the 95% CI. The diamond represents the pooled effect size (Hedges’ *g*). The vertical line at 0 represents the line of no effect; if the horizontal line of a study crosses this line, the effect of that study is not statistically significant. A Hedges’ *g*‐ value and its 95% CI entirely greater than 0 indicates higher NSE levels in the PSD group compared with controls (i. e., increased concentration in PSD), whereas values less than 0 indicate the opposite direction. CI, confidence interval; NSE, neuron‐specific enolase; PSD, post‐stroke depression.

A significant degree of heterogeneity was observed among the nine included studies (*I* ^2^ = 95.213, *p* < 0.001), which warranted the use of a random‐effects model. Utilizing this model, pooled Hedges’ *g* along with 95% CIs were calculated. It was found that PSD patients had notably elevated NSE levels in their peripheral blood compared to those of the controls. (Hedges’ *g* = 1.564, 95% CI: 0.938–2.191, *p* < 0.001) (Figure 4).

Due to the significant heterogeneity that reduces the reliability of the aggregated results, we attempted to use the extracted information for subgroup analysis. First of all, significant differences were identified in serum NSE levels between PSD patients and acute ischemic stroke (AIS) controls (Hedges’ *g* = 1.181, 95% CI: 0.517–1.844, *p* < 0.001) (Supporting Information Figure S9). The similar significant differences were observed in the subgroup of healthy controls (Hedges’ *g* = 3.226, 95% CI: 0.461–5.992, *p* = 0.022) (Supporting Information Figure S10). Nevertheless, heterogeneity among studies remained largely unchanged in both the AIS subgroup (*I* ^2^ = 94.309, *p* < 0.001) and the healthy control subgroup (*I* ^2^ = 92.391, *p* < 0.001). Additionally, depending on the detection method, although a pooled ES indicated higher peripheral blood NSE levels in PSD patients compared to controls in both the ELISA (Hedges’ *g* = 1.643, 95% CI: 0.581–2.705, *p* = 0.002) (Supporting Information Figure S11) and ECLIA (Hedges’ *g* = 2.061, 95% CI: 1.564–2.557, *p* < 0.001) (Supporting Information Figure S12) groups, heterogeneity did not decrease significantly in the ELISA subgroup (*I* ^2^ = 97.124, *p* < 0.001), while the ECLIA subgroup still exhibited moderate to high heterogeneity (*I* ^2^ = 54.792, *p* = 0.137). These findings suggest that the type of controls and assay methods may not be a significant factor influencing the source of heterogeneity (Table 2).

#### 3.3.2. Sensitivity Analysis

We performed a sensitivity analysis to evaluate the effect of each study on the combined Hedges’ *g* by sequentially excluding each study from the analysis. The association between peripheral NSE and PSD remained largely unaffected (Supporting Information Figure S13), indicating that no single study significantly altered the findings.

#### 3.3.3. Publication Bias

**Figure 5:**
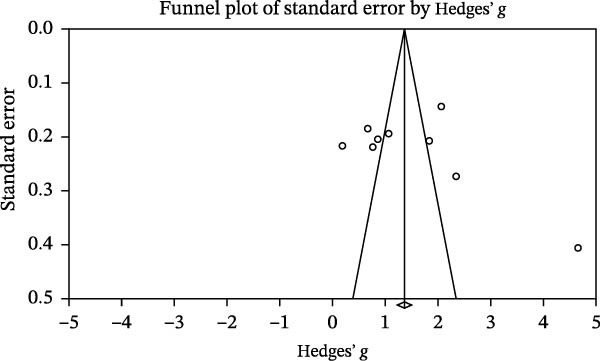
Funnel plot of precision using Hedges’ *g* statistics on PSD. Each dot represents an individual study included in the meta‐ analysis. The vertical line represents the pooled effect estimate from the meta‐analysis. The inverted dashed lines outline the expected 95% confidence interval (CI) for the distribution of studies in the absence of bias. The symmetrical distribution of various studies on PSD patients suggests no evident publication bias.

The publication bias was assessed using Egger’s test, and the corresponding funnel plot is presented in Figure 5. Our meta‐analysis revealed no significant publication bias, a finding further supported according to the quantitative results of Egger’s test (*p* = 0.411). Moreover, using the traditional fail‐safe N method, we determined that 960 additional studies would be necessary to alter the *p*‐ value to below 0.05. Thus, the association between peripheral blood NSE and PSD appears to be robust and unlikely to be impacted by the publication bias.

## 4. Discussion

As far as we know, this is the first meta‐analysis to systematically evaluate the existing evidence regarding the association between serum NSE levels and depression. Our quantitative assessment incorporated 18 eligible clinical studies. We tried to evaluate the association of depression with alterations in serum NSE levels. The primary outcome of this meta‐analysis revealed that serum NSE concentrations were significantly elevated in both MDD patients and PSD patients relative to matched controls. To minimize potential bias from individual studies, sensitivity analyses were conducted separately for the two themes, indicating that no individual study could overturn the established correlation. Publication bias may affect result validity by potentially suppressing false‐negative findings or amplifying false‐positive effects; however, our results indicated no significant publication bias in either part of this study, further supporting the robustness of our findings. Over the past few decades, identifying objective diagnostic markers for depression has remained a persistent challenge. Whether NSE, as a potential biomarker, is indeed associated with the onset and progression of depression remains a critical question. Although this clinical evidence gives us a novel direction for understanding the pathogenesis of depression, its considerable heterogeneity in the study limits the reliability of the evidence. To address this, we did subgroup analyses to find potential sources of heterogeneity. Subgroup analyses based on control type, country, diagnostic criteria, and assay method demonstrated that, among MDD patients, the significant difference in NSE levels persisted in most subgroups except for the Turkey, DSM ‐IV, and ECLIA subgroups. For PSD patients, the significant difference remained in all subgroups. However, despite these subgroup analyses, the sources of heterogeneity remain uncertain.

Based on the available information, there were certain data limitations in this study, such as a modest sample size, limited geographic coverage, and a limited scope of depression subtypes. These factors may serve as potential sources of heterogeneity and, to some extent, undermine the credibility and robustness of our findings, thereby limiting their generalizability across different regions and populations. Of note, existing epidemiological evidence consistently indicates that the incidence of depression is characterized by racial –ethnic disparities as well as geographic variations [39, 40]. For instance, a meta‐analysis of PSD prevalence in African countries found that Central Africa had the highest pooled estimate (50.92%, 95% CI: 45.94–55.90) in regional subgroup analyses, followed by West Africa [41]. Likewise, a cross‐national epidemiological study demonstrated that the lifetime and 12‐month prevalence estimates of MDD varied significantly across different countries [42]. Therefore, it is necessary to conduct further clinical studies covering more different depression subtypes and diverse population cohorts, which could provide new perspectives and evidence for a deeper understanding of the relevant issues.

What is the role of elevated NSE levels in depression? Numerous studies have confirmed that MDD is strongly associated with inflammation in the central nervous system [43]. On the other hand, PSD, as depressive symptoms that occur after a stroke in an individual, has been confirmed by both animal and clinical experiments to be closely related to neuroinflammation [44]. Specifically, in the neuroinflammatory environment of the depression patients’ brain, overactivated microglia releases abundant harmful inflammatory mediators that directly lead to neuronal injury [45]. Meanwhile, owing to the insufficient anti‐inflammatory capacity of astrocytes, a chronic inflammatory environment gradually develops, which further damages neurons and synapses through oxidative stress [46]. Notably, it has been shown that NSE can be released into the peripheral blood upon neuronal damage. Numerous clinical studies have identified NSE as a potential marker of neuroinflammation. For instance, a clinical trial investigating neuroinflammation following severe brain injury demonstrated that NSE exhibited a more significant association with inflammatory factors, such as IL‐6, compared to S100β [47]. Meanwhile, basic research affirms the value of NSE as a promising biomarker in neuropsychiatric disorders accompanied by inflammation [48]. Thus, it can be inferred that neuron damage caused by neuroinflammation is responsible for elevated peripheral blood NSE levels in depression.

However, the inherent pathophysiological complexity of depression also makes it impossible to diagnose depression solely on the basis of one biomarker but rather on combinations of multiple indicators. Therefore, it is necessary to identify a set of biomarkers associated with NSE. First, in the field of neuropsychiatric disorders related to neuronal damage, S100β and NSE are often studied together due to their close relationship in mechanisms. S100β reflects the level of CNS damage and is a specific marker for the diagnosis of brain damage [49]. One clinical study found that levels of both NSE and S100β increased together are substantially high in the plasma of people with MDD compared with healthy control individuals, with substantial ESs (Hedges’ *g* = 2.035 and 1.397, respectively) [22]. Moreover, there is a systematic review about the clinical utility of the biomarkers in the diagnosis of MDD, revealing that miRNAs in peripheral blood mononuclear cells also had significant diagnostic value [50]. Furthermore, studies on potential biomarkers in peripheral blood have found that MYT1 and combination of dopamine, GABA, tyramine, and kynuramine show high sensitivity in detecting MDD and thus have good practicality as variables [51]. In summary, future research should move beyond repeated validation of individual biomarkers. Instead, integrated multiomics and multimodal biomarker models represent a necessary direction toward achieving objective and precise diagnosis of depression.

This meta‐analysis has certain inherent limitations that should be acknowledged. First, due to the moderate sample size, the robustness and quality of the evidence may be affected. We observed heterogeneity in the association between peripheral NSE levels and depression. Although we conducted subgroup analyses according to the extracted data, the reason for this heterogeneity is still unclear. Due to the limitations of original data, we were unable to assess secondary outcomes, such as whether changes in NSE levels correlate with depression severity. Specifically, most included studies did not report stratified data based on the depression scale scores. Therefore, additional studies with larger sample sizes that also report severity scoring data are necessary to confirm and extend our results in the future. Second, the scope of the included studies on depression is limited. Through systematic searches, we found that high‐quality evidence on serum NSE levels in depression is almost exclusively concentrated in patients with MDD and PSD. In contrast, other depressive disorders, such as dysthymia, bipolar depression, or perinatal depression, lack a sufficient number of qualified studies. Therefore, further clinical research is needed to explore the connection between NSE in peripheral blood and other depression types. Third, there are still numerous possible methodological and clinical confounding factors, including age and gender distribution, methods of sample collection and processing, storage conditions, testing protocols, and geographical variations in sample sources. For example, all included studies on PSD were conducted in China. Consequently, the generalizability of our findings to other global populations may be constrained by these specific cultural background, healthcare system, and demographic contexts. Future studies are urgently needed to validate these results across diverse ethnic and cultural groups. Fourth, the clinical relevance of elevated peripheral NSE levels needs to be considered from multiple perspectives. It is not yet clear whether these increased peripheral NSE levels are a cause or a consequence of depression. Research has often demonstrated a positive association between NSE levels and infarct volume in AIS patients, but some studies have not supported this relationship [12]. While the role of NSE in reflecting the severity of cerebral infarction remains debated, these discrepancies may influence the interpretation of our findings. Therefore, further basic experimental research is necessary to clarify the relationship between poststroke craniocerebral injury and NSE secretion.

## 5. Conclusion

In summary, our findings suggest that elevated serum NSE levels may be associated with MDD or PSD. However, the value of NSE as a standalone diagnostic biomarker is limited. Future research should prioritize well‐powered randomized or adaptive trials to further validate the clinical role of NSE in the depression diagnosis and in combinatorial biomarker models. Additionally, larger and more representative clinical studies as well as preclinical animal models are needed to validate the efficacy and clarify the underlying mechanisms.

## Author Contributions

Ling Yang, Jingxuan Zhang, and Junwei Gao were involved in the study design. Qianbing Ge, Yangke Li, Tianye Sun, and Junwei Gao did the literature search, data collection and statistical analysis. Qianbing GeYangke Li, Tianye Sun, Yongbo Tan, and Xingru Wu were involved in drafting figures. Qianbing Ge, Yangke Li, Tianye Sun, Yongbo Tan, Chuanyu Wu, Jiao Zou, Ling Yang, Jingxuan Zhang, and Junwei Gao were involved in writing the paper and editing the language.

## Funding

No funding was received for this research.

## Disclosure

All authors contributed to critical revisions and approved the final version of the manuscript.

## Conflicts of Interest

The authors declare no conflicts of interest.

## Supporting Information

Additional supporting information can be found online in the Supporting Information section.

## Supporting information


**Supporting Information** Figure S1: Forest plot for the random‐effect meta‐analysis of the healthy subgroup on MDD. Figure S2: Forest plot for the random‐effect meta‐analysis of the China subgroup on MDD. Figure S3: Forest plot for the random‐effect meta‐analysis of the Turkey subgroup on MDD. Figure S4: Forest plot for the random‐effect meta‐analysis of the ELISA subgroup on MDD. Figure S5: Forest plot for the random‐effect meta‐analysis of the ECLIA subgroup on MDD. Figure S6: Forest plot for the random‐effect meta‐analysis of the DSM‐IV subgroup on MDD. Figure S7: Forest plot for the random‐effect meta‐analysis of the CCMD‐3 subgroup on MDD. Figure S8: Sensitivity analysis on MDD. Figure S9: Forest plot for the random‐effect meta‐analysis of the AIS subgroup on PSD. Figure S10: Forest plot for the random‐effect meta‐analysis of the healthy subgroup on PSD. Figure S11: Forest plot for the random‐effect meta‐analysis of the ELISA subgroup on PSD. Figure S12: Forest plot for the random‐effect meta‐analysis of the ECLIA subgroup on PSD. Figure S13: Sensitivity analysis on PSD.

## Data Availability

The data that support the findings of this study are available upon request from the corresponding author. The data are not publicly available due to privacy or ethical restrictions.
