# Supplementary material for: Aberrations in Peripheral Blood Neuron‐Specific Enolase Levels in Depression: A Systematic Review and Meta‐Analysis
Source: Depress Anxiety. 2026 Jul 13;2026:7759663. doi: 10.1155/da/7759663 (PMC13365796; doi:10.1155/da/7759663)
Supplement: Supplementary file 1 — Supporting Information Figure S1: Forest plot for the random‐effect meta‐analysis of the healthy subgroup on MDD. Figure S2: Forest plot for the random‐effect meta‐analysis of the China subgroup on MDD. Figure S3: Forest plot for the random‐effect meta‐analysis of the Turkey subgroup on MDD. Figure S4: Forest plot for the random‐effect meta‐analysis of the ELISA subgroup on MDD. Figure S5: Forest plot for the random‐effect meta‐analysis of the ECLIA subgroup on MDD. Figure S6: Forest plot for the random‐effect meta‐analysis of the DSM‐IV subgroup on MDD. Figure S7: Forest plot for the random‐effect meta‐analysis of the CCMD‐3 subgroup on MDD. Figure S8: Sensitivity analysis on MDD. Figure S9: Forest plot for the random‐effect meta‐analysis of the AIS subgroup on PSD. Figure S10: Forest plot for the random‐effect meta‐analysis of the healthy subgroup on PSD. Figure S11: Forest plot for the random‐effect meta‐analysis of the ELISA subgroup on PSD. Figure S12: Forest plot for the random‐effect meta‐analysis of the ECLIA subgroup on PSD. Figure S13: Sensitivity analysis on PSD. [file DA-2026-7759663-s001.docx]

**
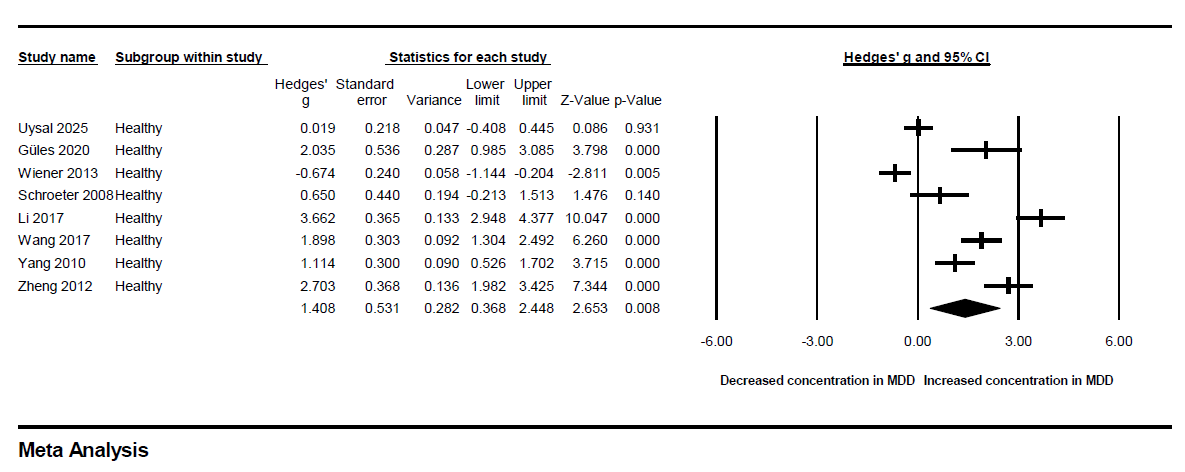
**

**Supplementary Figure 1**: Forest plot for the random-effect meta-analysis of the healthy subgroup on MDD. The overall effect estimate for the MDD showed significantly elevated NSE levels. Each black box represents a study’s point estimate, and the horizontal line shows the 95%CI. The diamond represents the pooled effect size (Hedges’ g). The vertical line at 0 represents the line of no effect; if the horizontal line of a study crosses this line, the effect of that study is not statistically significant. A Hedges' g value and its 95% CI entirely greater than 0 indicates higher NSE levels in the MDD group compared with healthy subgroups (i.e., increased concentration in MDD), whereas values less than 0 indicate the opposite direction. MDD: major depressive disorder; CI: confidence interval.


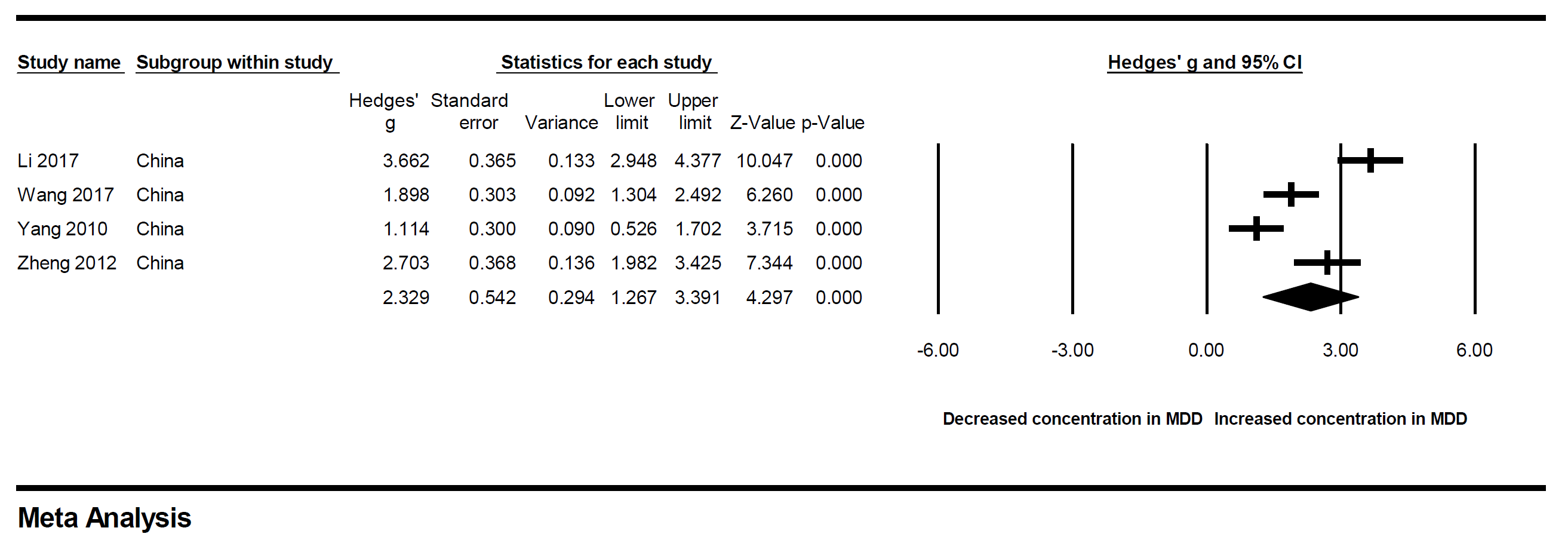
**Supplementary Figure 2**: Forest plot for the random-effect meta-analysis of the China subgroup on MDD. The overall effect estimate for the MDD showed significantly elevated NSE levels. Each black box represents a study’s point estimate, and the horizontal line shows the 95%CI. The diamond represents the pooled effect size (Hedges’ g). The vertical line at 0 represents the line of no effect; if the horizontal line of a study crosses this line, the effect of that study is not statistically significant. A Hedges' g value and its 95% CI entirely greater than 0 indicates higher NSE levels in the MDD group compared with China subgroups (i.e., increased concentration in MDD), whereas values less than 0 indicate the opposite direction. MDD: major depressive disorder; CI: confidence interval.


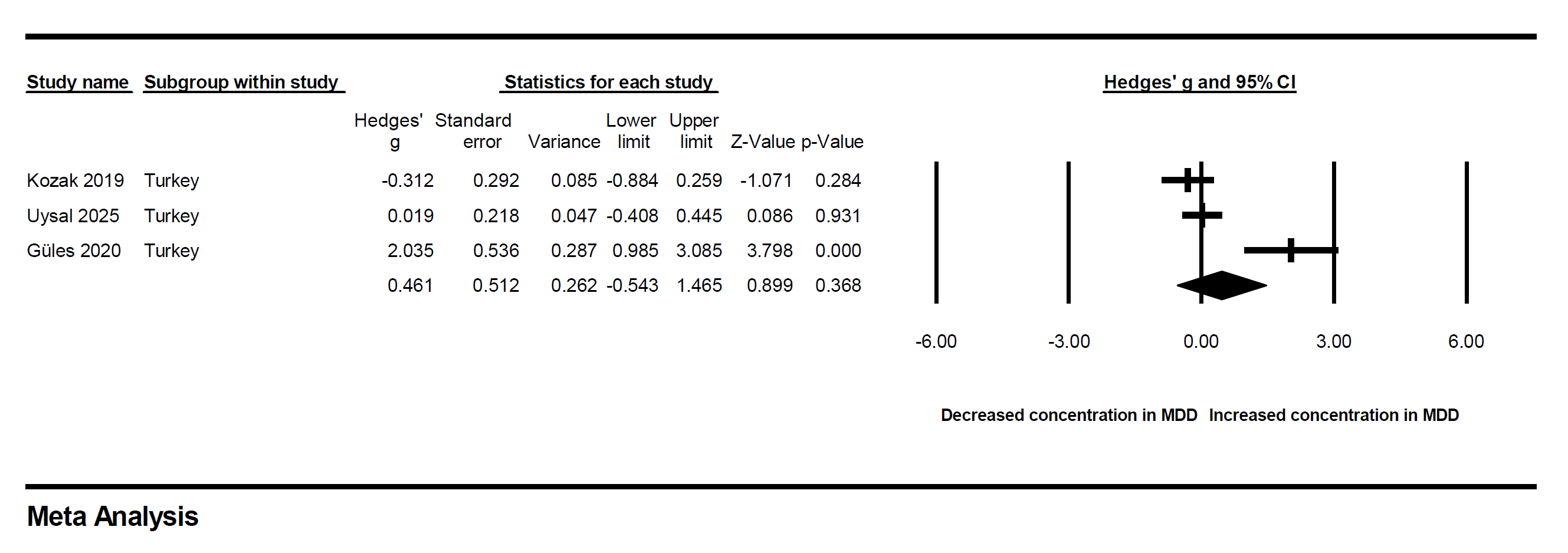
 **Supplementary Figure 3**: Forest plot for the random-effect meta-analysis of the Turkey subgroup on MDD. The overall effect estimate showed no significantly elevated NSE levels. Each black box represents a study’s point estimate, and the horizontal line shows the 95%CI. The diamond represents the pooled effect size (Hedges’ g). The vertical line at 0 represents the line of no effect; if the horizontal line of a study crosses this line, the effect of that study is not statistically significant. A Hedges' g value and its 95% CI entirely greater than 0 indicates higher NSE levels in the MDD group compared with Turkey subgroups (i.e., increased concentration in MDD), whereas values less than 0 indicate the opposite direction. MDD: major depressive disorder; CI: confidence interval.


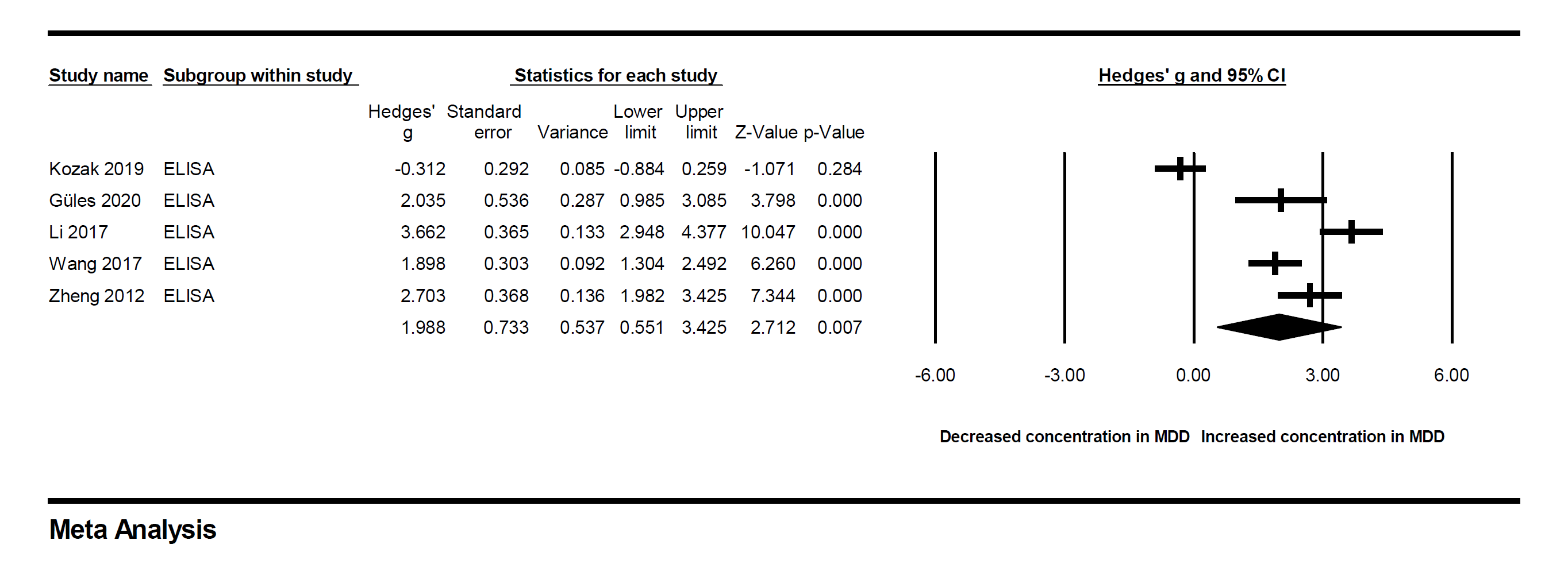
**Supplementary Figure 4**: Forest plot for the random-effect meta-analysis of the ELISA subgroup on MDD. The overall effect estimate for the MDD showed significantly elevated NSE levels. Each black box represents a study’s point estimate, and the horizontal line shows the 95%CI. The diamond represents the pooled effect size (Hedges’ g). The vertical line at 0 represents the line of no effect; if the horizontal line of a study crosses this line, the effect of that study is not statistically significant. A Hedges' g value and its 95% CI entirely greater than 0 indicates higher NSE levels in the MDD group compared with ELISA subgroups (i.e., increased concentration in MDD), whereas values less than 0 indicate the opposite direction. MDD: major depressive disorder; CI: confidence interval.


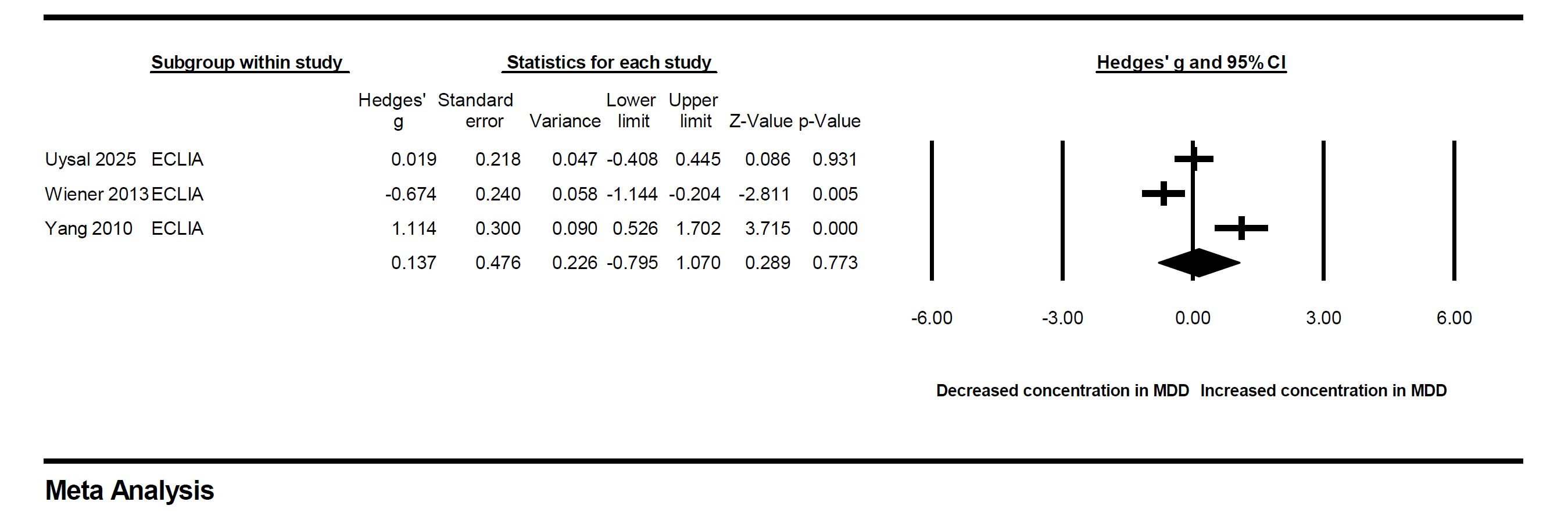
**Supplementary Figure 5**: Forest plot for the random-effect meta-analysis of the ECLIA subgroup on MDD. The overall effect estimate showed no significantly elevated NSE levels. Each black box represents a study’s point estimate, and the horizontal line shows the 95%CI. The diamond represents the pooled effect size (Hedges’ g). The vertical line at 0 represents the line of no effect; if the horizontal line of a study crosses this line, the effect of that study is not statistically significant. A Hedges' g value and its 95% CI entirely greater than 0 indicates higher NSE levels in the MDD group compared with ECLIA subgroups (i.e., increased concentration in MDD), whereas values less than 0 indicate the opposite direction. MDD: major depressive disorder; CI: confidence interval.


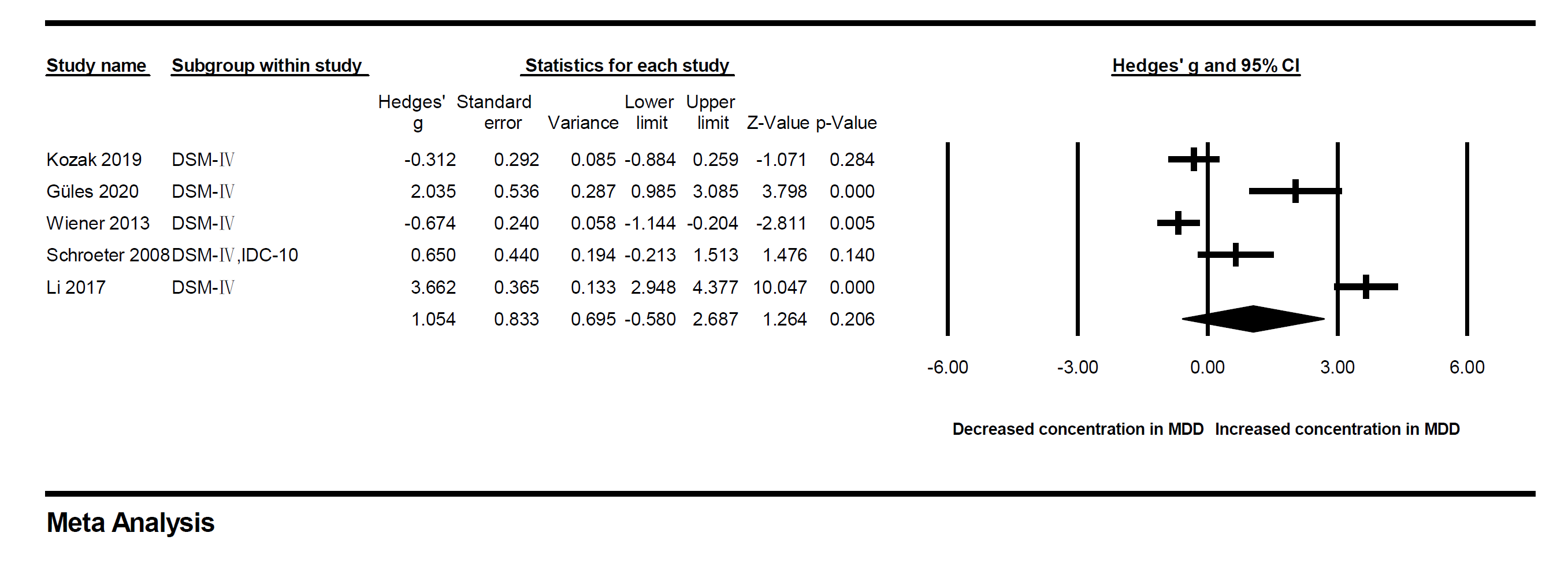
**Supplementary Figure 6**: Forest plot for the random-effect meta-analysis of the DSM-Ⅳ subgroup on MDD. The overall effect estimate showed no significantly elevated NSE levels. Each black box represents a study’s point estimate, and the horizontal line shows the 95%CI. The diamond represents the pooled effect size (Hedges’ g). The vertical line at 0 represents the line of no effect; if the horizontal line of a study crosses this line, the effect of that study is not statistically significant. A Hedges' g value and its 95% CI entirely greater than 0 indicates higher NSE levels in the MDD group compared with DSM-Ⅳ subgroups (i.e., increased concentration in MDD), whereas values less than 0 indicate the opposite direction. MDD: major depressive disorder; CI: confidence interval.


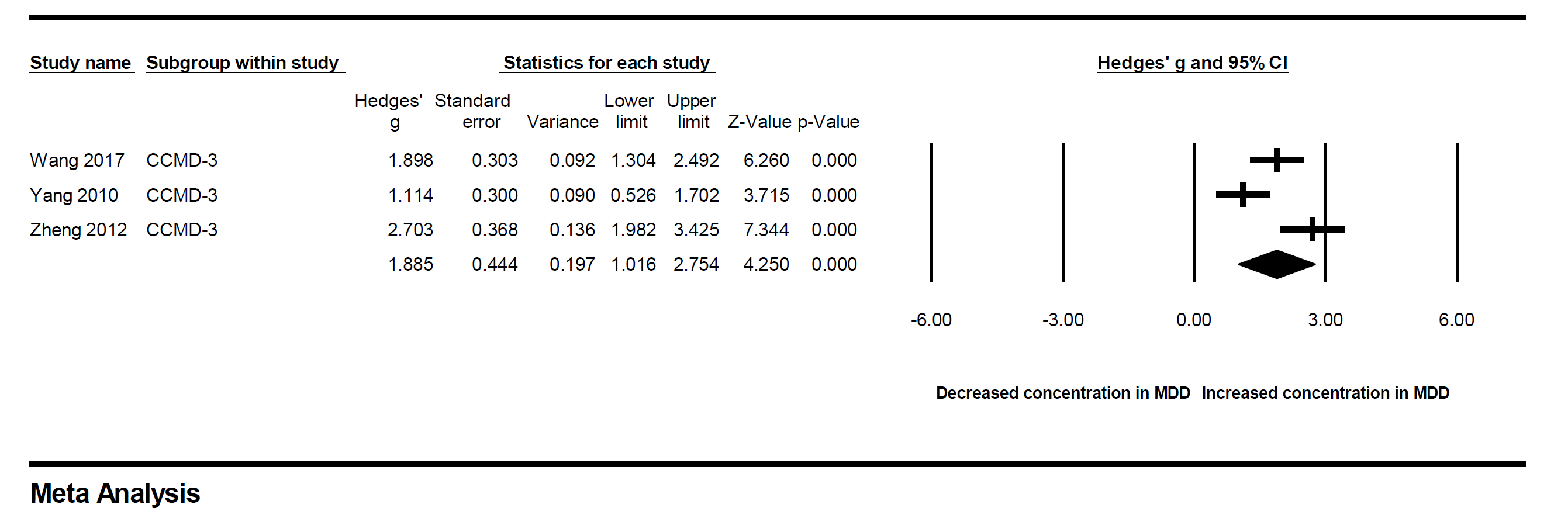
**Supplementary Figure 7**: Forest plot for the random-effect meta-analysis of the CCMD-3 subgroup on MDD. The overall effect estimate for the MDD showed significantly elevated NSE levels. Each black box represents a study’s point estimate, and the horizontal line shows the 95%CI. The diamond represents the pooled effect size (Hedges’ g). The vertical line at 0 represents the line of no effect; if the horizontal line of a study crosses this line, the effect of that study is not statistically significant. A Hedges' g value and its 95% CI entirely greater than 0 indicates higher NSE levels in the MDD group compared with CCMD-3 subgroups (i.e., increased concentration in MDD), whereas values less than 0 indicate the opposite direction. MDD: major depressive disorder; CI: confidence interval.


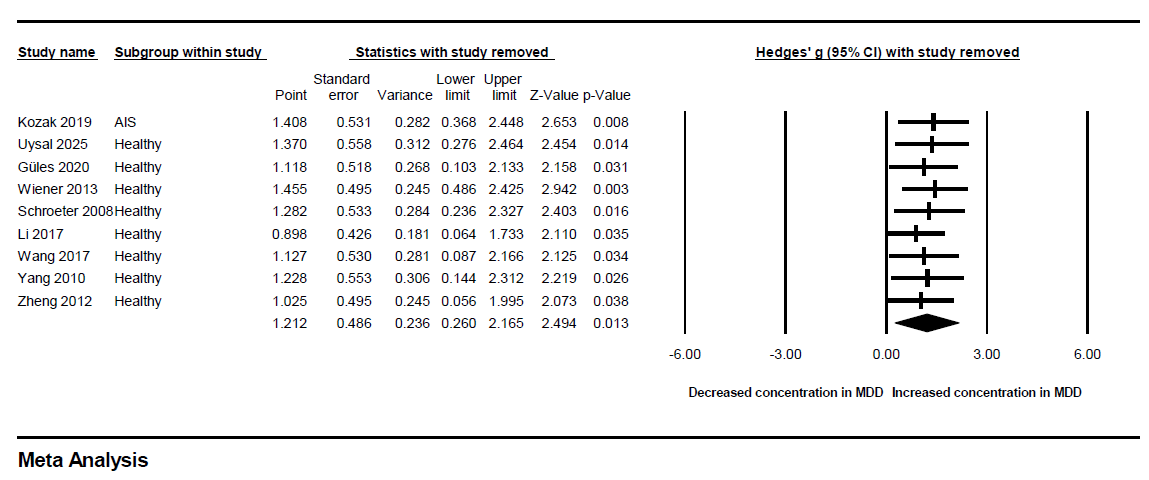


**Supplementary Figure 8**: Sensitivity analysis on MDD. Each black box represents the pooled effect size of the remaining studies after the exclusion of that particular study, and the horizontal line indicates the 95%CI. If excluding a study does not significantly change the pooled effect size, the meta-analysis results are stable. Conversely, large differences or opposite conclusions suggest poor stability. MDD: major depressive disorder; CI: confidence interval.


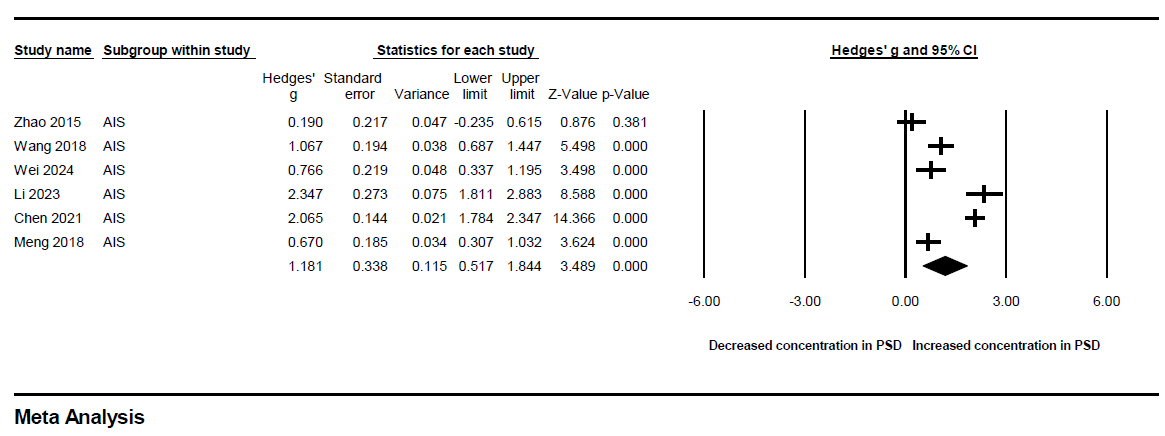


**Supplementary Figure 9**: Forest plot for the random-effect meta-analysis of the AIS subgroup on PSD. The overall effect estimate for the PSD showed significantly elevated NSE levels. Each black box represents a study’s point estimate, and the horizontal line shows the 95%CI. The diamond represents the pooled effect size (Hedges’ g). The vertical line at 0 represents the line of no effect; if the horizontal line of a study crosses this line, the effect of that study is not statistically significant. A Hedges’ g value and its 95% CI entirely greater than 0 indicates higher NSE levels in the PSD group compared with AIS subgroups (i.e., increased concentration in PSD), whereas values less than 0 indicate the opposite direction. PSD: post-stroke depression; CI: confidence interval.


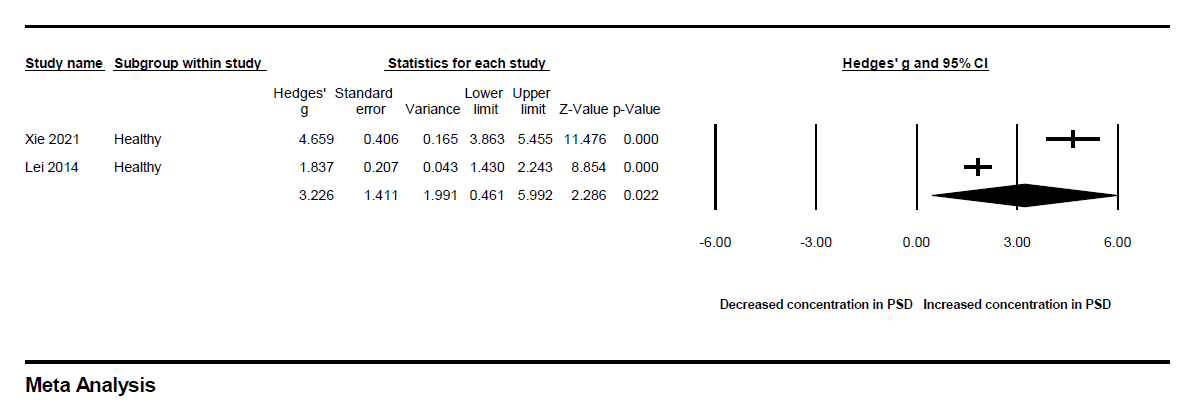


**Supplementary Figure 10**: Forest plot for the random-effect meta-analysis of the healthy subgroup on PSD. The overall effect estimate for the PSD showed significantly elevated NSE levels. Each black box represents a study’s point estimate, and the horizontal line shows the 95%CI. The diamond represents the pooled effect size (Hedges’ g). The vertical line at 0 represents the line of no effect; if the horizontal line of a study crosses this line, the effect of that study is not statistically significant. A Hedges’ g value and its 95% CI entirely greater than 0 indicates higher NSE levels in the PSD group compared with healthy subgroups (i.e., increased concentration in PSD), whereas values less than 0 indicate the opposite direction. PSD: post-stroke depression; CI: confidence interval.


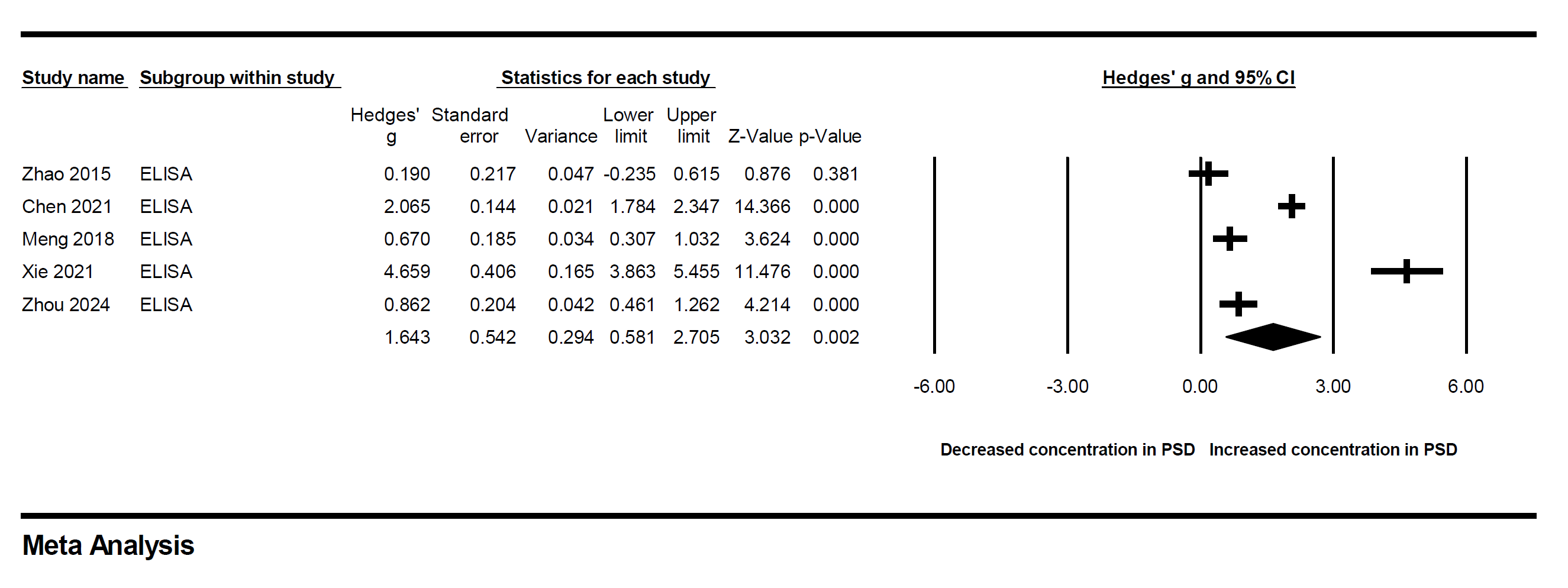
**Supplementary Figure 11**: Forest plot for the random-effect meta-analysis of the ELISA subgroup on PSD. The overall effect estimate for the PSD showed significantly elevated NSE levels. Each black box represents a study’s point estimate, and the horizontal line shows the 95%CI. The diamond represents the pooled effect size (Hedges’ g). The vertical line at 0 represents the line of no effect; if the horizontal line of a study crosses this line, the effect of that study is not statistically significant. A Hedges’ g value and its 95% CI entirely greater than 0 indicates higher NSE levels in the PSD group compared with ELISA subgroups (i.e., increased concentration in PSD), whereas values less than 0 indicate the opposite direction. PSD: post-stroke depression; CI: confidence interval.


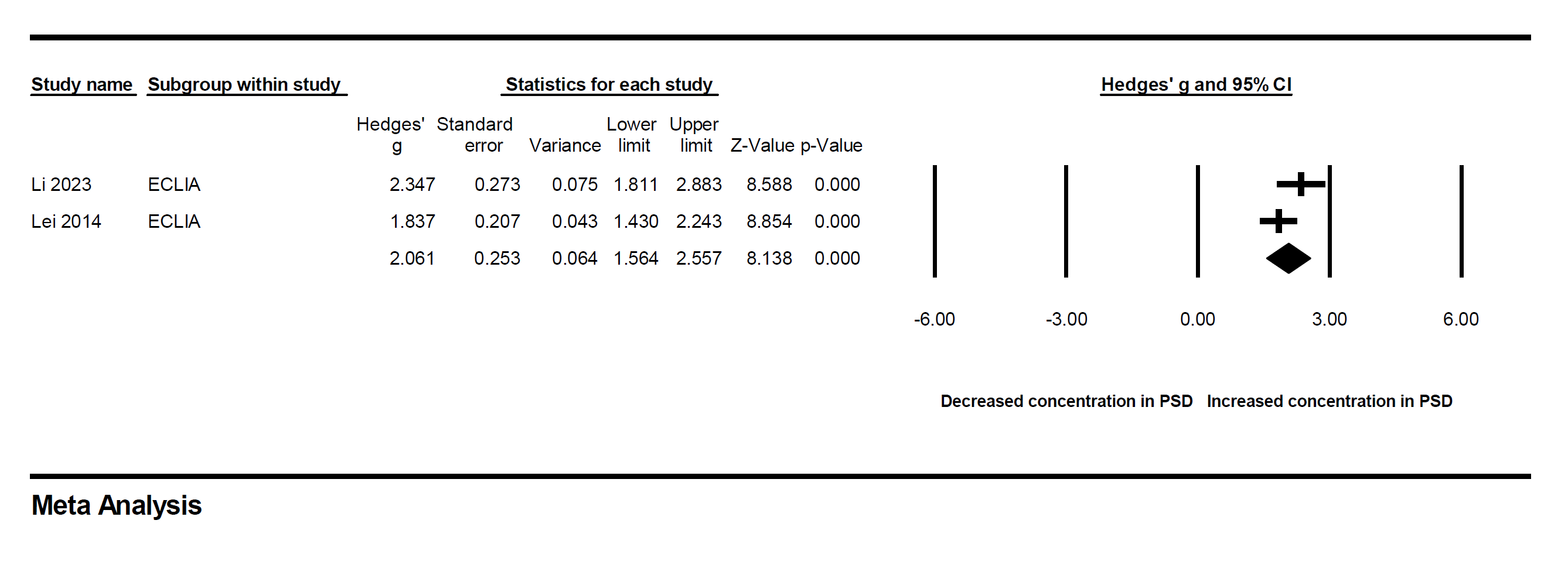
**Supplementary Figure 12**: Forest plot for the random-effect meta-analysis of the ECLIA subgroup on PSD. The overall effect estimate for the PSD showed significantly elevated NSE levels. Each black box represents a study’s point estimate, and the horizontal line shows the 95%CI. The diamond represents the pooled effect size (Hedges’ g). The vertical line at 0 represents the line of no effect; if the horizontal line of a study crosses this line, the effect of that study is not statistically significant. A Hedges’ g value and its 95% CI entirely greater than 0 indicates higher NSE levels in the PSD group compared with ECLIA subgroups (i.e., increased concentration in PSD), whereas values less than 0 indicate the opposite direction. PSD: post-stroke depression; CI: confidence interval.


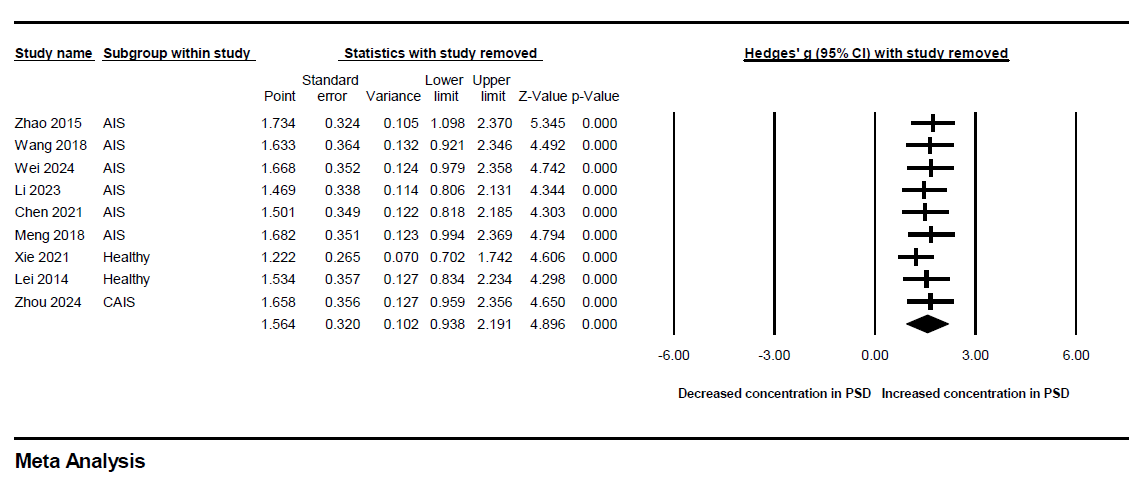


**Supplementary Figure 13**: Sensitivity analysis on PSD. Each black box represents the pooled effect size of the remaining studies after the exclusion of that particular study, and the horizontal line indicates the 95%CI. If excluding a study does not significantly change the pooled effect size, the meta-analysis results are stable. Conversely, large differences or opposite conclusions suggest poor stability. PSD: post-stroke depression; CI: confidence interval.
